# Sweet Selenium: Synthesis and Properties of Selenium-Containing Sugars and Derivatives

**DOI:** 10.3390/ph13090211

**Published:** 2020-08-26

**Authors:** Francesca Mangiavacchi, Italo Franco Coelho Dias, Irene Di Lorenzo, Pawel Grzes, Martina Palomba, Ornelio Rosati, Luana Bagnoli, Francesca Marini, Claudio Santi, Eder Joao Lenardao, Luca Sancineto

**Affiliations:** 1 Group of Catalysis, Synthesis and Organic Green Chemistry, Department of Pharmaceutical Sciences, University of Perugia, Via del Liceo 1, 06100 Perugia (PG), Italy; italo@chemist.com (I.F.C.D.); iry.arg@gmail.com (I.D.L.); martina.palomba@unipg.it (M.P.); ornelio.rosati@unipg.it (O.R.); luana.bagnoli@unipg.it (L.B.); francesca.marini@unipg.it (F.M.); claudio.santi@unipg.it (C.S.); 2 Laboratory of Clean Organic Synthesis, Federal University of Pelotas, Pelotas 96010-900, Brazil; lenardao@ufpel.edu.br; 3 Department of Chemistry, University of Białystok, ul. Ciołkowskiego 1K, 15-245 Białystok, Poland; pawel.grzes1993@gmail.com

**Keywords:** selenium, sugar, selenoglycosides, water-soluble

## Abstract

In the last decades, organoselenium compounds gained interest due to their important biological features. However, the lack of solubility, which characterizes most of them, makes their actual clinical exploitability a hard to reach goal. Selenosugars, with their intrinsic polarity, do not suffer from this issue and as a result, they can be conceived as a useful alternative. The aim of this review is to provide basic knowledge of the synthetic aspects of selenosugars, selenonium salts, selenoglycosides, and selenonucleotides. Their biological properties will be briefly detailed. Of course, it will not be a comprehensive dissertation but an analysis of what the authors think is the cream of the crop of this interesting research topic.

## 1. Introduction

Organoselenium compounds are gaining interest in the medicinal chemistry community due to their promising biological activities for a wide range of clinical applications [1,2,3,4,5,6,7]. Starting from Ebselen, the first compound showing the ability to function as glutathione peroxidase (GPx) mimetic [8], several Se-bearing molecules were reported as anticancer [9,10,11,12], anti-inflammatory [13,14], antidepressant [15,16], and antibacterial [17,18,19], among others. Worth mentioning, some of us developed the DiSeBAs class of compounds [20], endowed with a potent anti-HIV activity due to their ability to inhibit the key viral protein NCp7 [21,22]. In the antiviral research field, Ebselen was recently discovered as the most potent inhibitor of the main protease belonging to the pandemic coronavirus SARS-Cov-2 [23].

The analysis of the physicochemical properties of investigational molecules is a pivotal aspect for scientists involved in designing a pharmacologically active compound. In particular, a certain degree of aqueous solubility is a feature that a compound needs to possess to become bioavailable when administered orally [24,25]. Even if organoselenium compounds do showcase important biological properties, their actual clinical exploitability could be hampered by the low aqueous solubility which precludes them from reaching therapeutically relevant doses in blood. A possible strategy to overcome this barrier is to combine the important pharmacological properties of selenium with the physicochemical properties of sugars, leading to the class of selenosugars and their derivatives, that are the object of the present review article. With this in mind, we aim to review the organoselenium compounds endowed with the highest aqueous solubility, at least from a chemical point of view. Most of the selenosugars developed to date will be herein detailed highlighting both the pharmacological and the synthetic aspects, in order to equip the reader with useful knowledge to introduce this useful building block in designed compounds.

## 2. Selenopyranes and Selenofuranes

**Figure 1:**
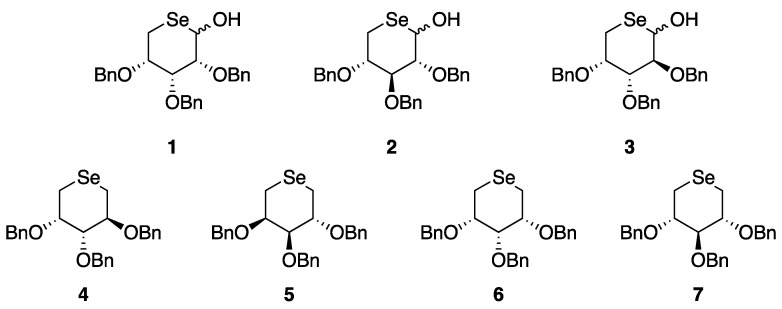
Structures of the 5-seleno-pentopyranose.

One of the first attempts to explore the synthesis of selenocarbohydrates is reported in the works of Schiesser’s group, collected in a recently published perspective [26]. Their interest was mainly focused on the synthesis of both selenopyranes and selenofuranes as potent water-soluble selenium derivatives able to act as potential antioxidants endowed with good oral bioavailability. Based on his experience on the selenium free-radical chemistry, Schiesser proposed the synthesis of 5-seleno-D-pentopyranose protected sugars **1–7** (Figure 1), using two different approaches [27].

**Scheme 1:**
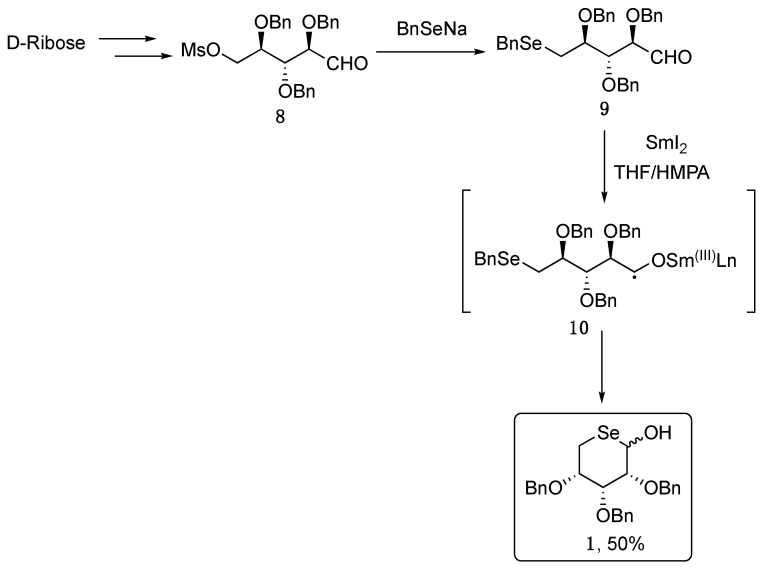
Synthesis of compound **1**.

As depicted in Scheme 1, compound **1** was successfully isolated as a mixture of anomers through an intramolecular homolytic substitution mediated by samarium(II) iodide, that triggers the formation of the radical intermediate **10** from the seleno-aldehyde **9**, which was in turn obtained from D-ribose [27].

**Scheme 2:**
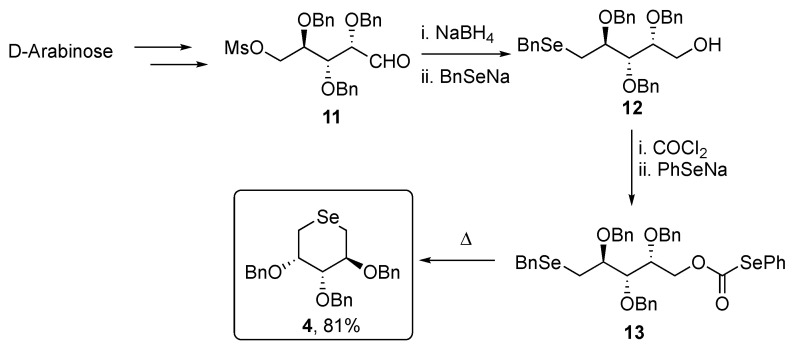
Synthesis of compound **4**.

The same strategy was applied for the synthesis of **2** and **3** starting from D-Xylose and D-Arabinose, respectively. On the other hand, compounds **4**–**7** were prepared by thermolysis of the selenoformate precursors, in which an intramolecular nucleophilic attack of the benzylselanyl group took place, followed by the expulsion of carbon dioxide. Compound **4** was prepared starting from D-arabinose, which was converted into 2,3,4-tri-*O*-benzyl-5-*O*-methanesulfonylarabinose **11** then reduced with NaBH_4_ and selenofunctionalized through the reaction with benzylselenolate, giving the selenide **12**. Compound **12** was then activated as selenocarbonate **13** and finally thermally converted into the target selenosugar **4**. The same synthetic strategy was adopted for the synthesis of **5** but starting from L-arabinose (Scheme 2).

**Scheme 3:**
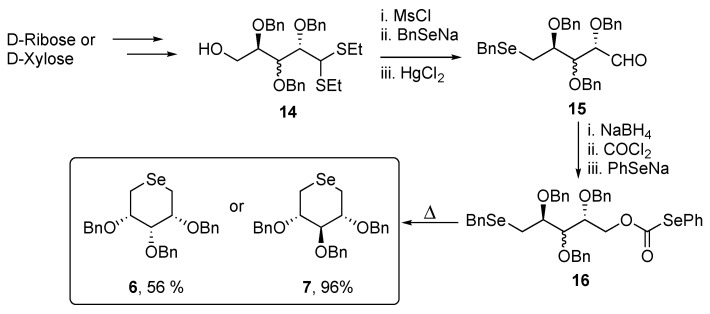
Synthesis of compounds **6** and **7**.

The synthesis of protected sugars **6** and **7** required a slight modification of the synthetic route to overcome stability issues related to D-ribose and D-xylose analogues of compound **11**, hence, the dithioacetals **14** was used instead of the unprotected aldehyde (Scheme 3).

**Figure 2:**
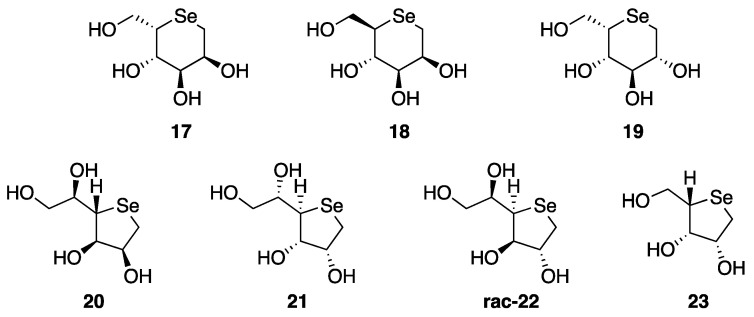
Structures of compounds **17**–**23**.

Unfortunately, all the above compounds were not isolated as free sugars due to several issues related to the removal of benzyl groups, which hampered the deprotected derivatives to be obtained. Compounds **17**–**23** (Figure 2) were instead prepared after the authors developed a different synthetic strategy.

**Scheme 4:**
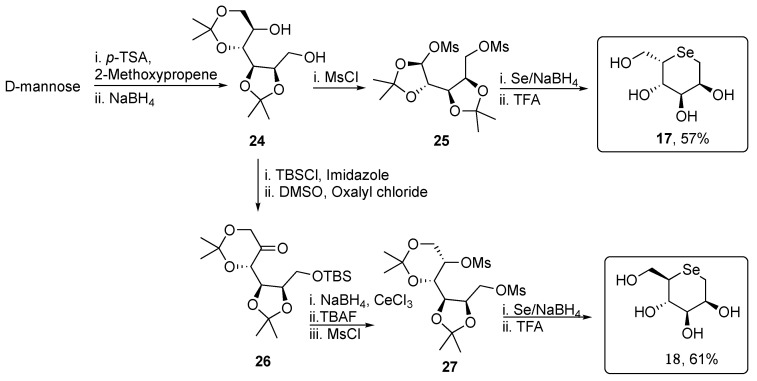
Synthesis of unprotected selenosugars **17** and **18**.

Compounds **17** and **18** were efficiently obtained starting from D-mannose, which was firstly regioselectively protected and then reduced, giving the diol **24**, that was successively activated by bismesylation for the subsequent selenium nucleophilic substitution, affording **17** after deprotection using TFA (Scheme 4). For the selenosugar **18**, a C-5 double inversion strategy was necessary. The diol **24** was quantitatively protected with TBSCl and then subjected to Swern oxidation, yielding **26** in nearly quantitative yield. The keto moiety was stereoselectively converted into the key alcohol by employing the Luche-type reductive conditions, and then activated as mesylate in compound **27**. Finally, its treatment with an ethanolic solution of Na_2_Se and the successive acidic deprotection gave the target selenosugar **18** [28].

**Scheme 5:**
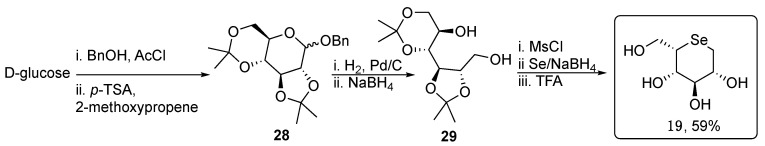
Synthesis of unprotected selenosugar **19**.

Similarly, seleno-L-gulitol **19** was obtained starting from D-glucose following a similar synthetic pathway, in which selenium is introduced as a nucleophile (Scheme 5) [29].

**Scheme 6:**
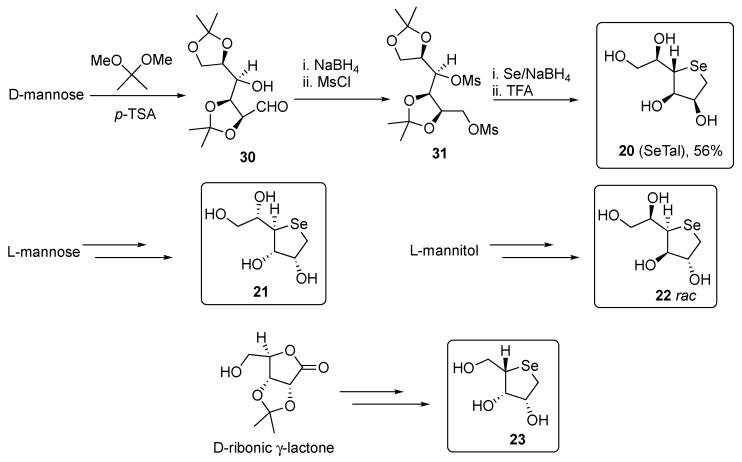
Synthesis of compounds **20**–**23**.

A freshly prepared solution of Na_2_Se in ethanol was used also for the preparation of 5-membered selenofurans **20**–**23**, as depicted in Scheme 6. As example, the synthesis of the pyranose 1,4-dideoxy-4-seleno-D-talitol **20**, began from D-mannose, which was reacted with 2,2-dimethoxypropane in the presence of catalytic *p*-toluenesulfonic acid to give the aldehyde **30**. The reduction of **30** with sodium borohydride in methanol, followed by reaction with MsCl gave the corresponding bismethanesulfonate intermediate **31**. Compound **31** was then converted into the protected selenosugar and finally deprotected by treatment with trifluoroacetic acid in dichloromethane [29].

Similarly to **20**, compounds **21**–**23** were obtained from L-mannose, L-mannitol, and D-ribonic γ-lactone, respectively, adopting the same synthetic protocol (Scheme 6) [26].

As previously alluded, the great interest in these compounds is related to their ability to act as scavengers against powerful biological oxidants such as hypohalous acids (HOX, X = Cl, Br) produced by mieloperoxidase (MPO) at inflammation sites, and peroxynitrous acid (ONOOH) produced by the activated macrophages. All the above-mentioned oxidants are part of the cellular arsenal defense against pathogens and at low concentrations act as a key signal for the innate immune response [30,31]. Therefore, their production is finely controlled and perfectly balanced. As these finely controlled defense mechanisms are perturbed, the so-called “oxidative stress” status occurs [32], leading to a number of human diseases such as asthma, metabolic disorders, cardiovascular diseases, some neurodegenerative conditions, just to cite some [33].

The second-order rate constant of sugars **17**–**20** with hypohalous acid has been determined to explore the ability of such compounds to protect sensitive biological targets (such as thiols, sulfides, proteins, nucleic acids, etc.) against oxidative damage. The 1,4-anhydro-4-seleno-D-talitol (SeTal, **20**) has revealed itself as a very interesting case study, showing good scavenger activity for several biological relevant oxidants (HOCl, HOBr, HOSCN, ONOOH) in vitro and in human plasma [29,34].

**Figure 3:**
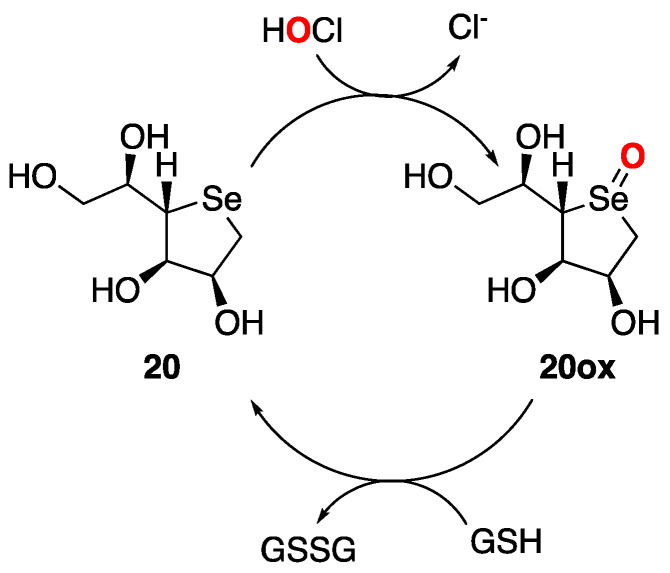
Catalytic cycle of 1,4-anhydro-4-seleno-D-talitol (SeTal) (**20**).

SeTal reacts removing these dangerous chemicals and turns into the corresponding selenoxyde derivative, which is then reduced back to selenide thanks to the abundant presence of cellular thiols, such as glutathione (GSH, Figure 3) [35]. Given its high antioxidant profile, SeTal (**20**) was further investigated regarding its potential therapeutic application in the context of the wound healing process.

Impaired wound healing is a key marker of type 2 diabetes, where endothelial disfunction is related to high glucose levels that triggers the formation of reactive oxygen species (ROS). In vitro studies have showed that SeTal (**20**) prevents high-glucose-induced endothelial dysfunction in mouse aorta, suggesting that this selenosugar is capable of repairing endothelial dysfunction brought on by the diabetic condition [36]. In light of this, compound **20** was tested for its ability to restore the wound healing process in vivo. Investigations carried out on diabetic mice showed a significant increase of wound healing from 40% to 80% after 10 days using a 1 mM solution of selenosugar. The wound-healing properties of SeTal were found to be superior to that of other water-soluble selenides [26].

Selenopyranoses, bioisosters of natural *O* or *N*-containing sugars, have been also investigated as glycosidase inhibitors. Glycosidases are a group of enzymes that catalyze the hydrolysis of glycosidic linkages degrading oligosaccharides and glycoconjugates [37]. Their broad activity makes them suitable targets for the development of anticancer, anti-type 2 diabetes, and antiviral agents [38].

**Scheme 7:**
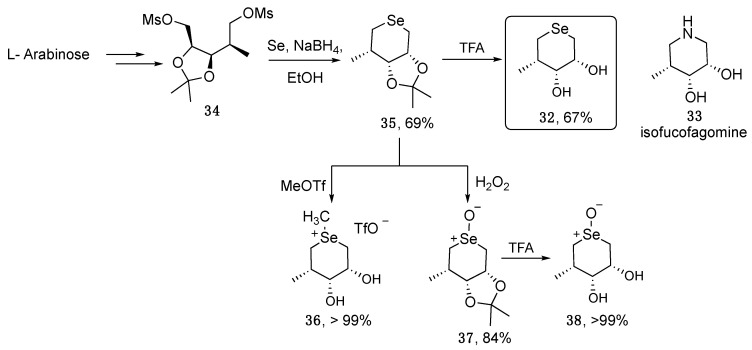
Synthesis of Se-analogues of isofucofagomine.

Recently, Merino-Montiel et al. reported the synthesis of compound **32** and tested its ability to act as a glycosidase inhibitor in analogy with the natural, potent inhibitor isofucofagomine **33**. Starting from L-arabinose, the synthetic strategy relies on the formation of the key activated intermediate **34**, which reacted with nucleophilic selenium generated in situ by reduction of elemental selenium with NaBH_4_ (Scheme 7). The authors were also interested in understanding how the oxidation state of selenium affects its biological activity, and therefore the methylated and oxidized derivatives **36** and **38** were also prepared. Compounds **32**, **36**, and **38** were investigated for their ability to inhibit α- and β-glucosidase as well as α-L-fucosidase. A beneficial effect of the positive charge on the selenium atom reflected by the higher activity of compound **36** with respect to **32** was observed. Furthermore, selenoxide **38**, although less active with respect to the natural iminosugar **33**, showed high selectivity for the α-L-fucosidase. Besides, the ability to mimic the antioxidant activity of Gluthathione Peroxidase (GPx) was also tested by evaluating the capacity of the protected compound **35** for scavenging hazardous ROS, such as hydrogen peroxide. When tested at 10 mol %, compound **35** displayed a T_50_ of 32 min [39].

## 3. Selenonium Salts

Selenonium salt derivatives of selenosugars are surely worth mentioning. They were initially prepared as a result of the pioneering work of Pinto and colleagues, who first developed the synthetic protocol for their preparation.

The interest of Pinto’s group was related to the development of selenium isosters of the active sulfonium salt derivatives, largely distributed in the plants of *Salacia* species. In particular, the root, leaf, and stem extracts from *Salacia reticulata* have been widely used in the Ayurvedic and traditional medicine for the treatment of diabetes and as a food supplement due to its ability to prevent obesity, especially in Japan and the USA [40,41]. Diabetes is a complex metabolic disease often associated with cardiovascular complications and other pathologies. The WHO (World Health Organization) has esteemed 60 million people affected by diabetes in the European area, with a large increase in different age groups. Type 2 diabetes is considered one of the main causes of morbidity and mortality worldwide which, unfortunately, is predicted to increase its spread, and more importantly, it is the fourth cause of death in most of the developed countries [42]. Therefore, the prevention, as well as the development of good therapeutic strategies able to control the high levels of glucose and all of the diabetes complications, are highly desirable.

**Figure 4:**
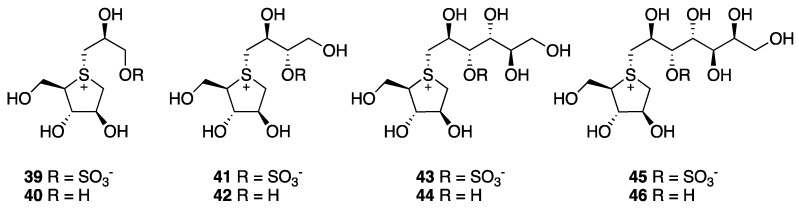
Structure of active compounds in *Salacia* species.

The active compounds from *Salacia* species are displayed in Figure 4. Salaprinol (**39**), salacinol (**41**), ponkoranol (**43**), kotalanol (**45**), and the corresponding de-*O*-sulfonated derivatives **40**, **42**, **44**, and **46** (Figure 3) have been correlated with the ability to modulate both lipidic and carbohydrate metabolism, in the latter case because of their glycosidases inhibitory property [41,43].

Over a decade, Pinto and co-workers largely contributed to the structural elucidation of the cited active components of *Salacia* species [44]. In addition, they widely explored the structural modifications of such compounds and the effect on the inhibitory activity of maltase-glucoamylase (MGAM) and sucrase-isomaltase (SI) [45,46,47]. Both MGAM and SI are intestinal α-glucosidase enzymes responsible for the hydrolysis of polysaccharides resulting in the glucose release and absorption, therefore considered suitable targets for the development of anti-diabetes drugs [48].

**Figure 5:**
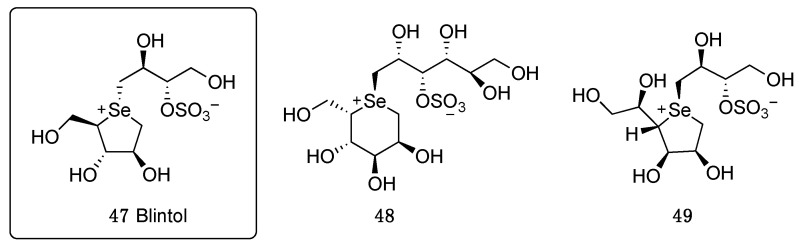
Structures of selenosugars **47–49**.

The introduction of selenium as a replacer of sulfur in the aldose skeleton of salacinol (compound **41**, Figure 4) resulted in blintol (**47**, Figure 5), which showed a really good inhibitory activity against human intestinal glucosidases. The activity was explained by a perfect superimposition of 1,4-anhydro-4-seleno-D-arabinitol ion with the oxacarbonium intermediate from the transition state of the glycoside bond breakage [49]. Starting from blintol, Pinto’s group has synthesized and investigated a great variety of derivatives that will be hereafter briefly summarized in two main groups:

- Modifications of the heterocyclic ring, and
- Modification on the polyhydroxylated chain.

### 3.1. Modification of the Heterocyclic Ring

The replacement of sulfur by the heavier chalcogen selenium positively affected the inhibition activity against glycosidase enzymes, showing also the ability to be selective on some of them. Indeed, compound **47** was capable of inhibiting three glycosidase enzymes, namely glucoamylase G2, porcine pancreatic R-amylase, and barley R-amylase, beside the human MGAM [50,51]. The synthesis and investigation of the six-member analogue, 1,5-anhydro-1-seleno-L-gulitol derivative **48** and a different heterocycle configuration, 1,4-anhydro-1-seleno-D-talitol **49**, was also reported (Figure 5).

Worth mentioning, the ring-expansion negatively affected the inhibitory activity. As a result, compound **48** is a worse glycosidase inhibitor than **47**, highlighting that the five-member ring is a key structural element for the activity. Furthermore, it has been demonstrated that the configuration of the D-arabinitol ring is another key feature, determinant for the activity [52,53].

### 3.2. Modification on the Polyhydroxylated Chain

**Figure 6:**
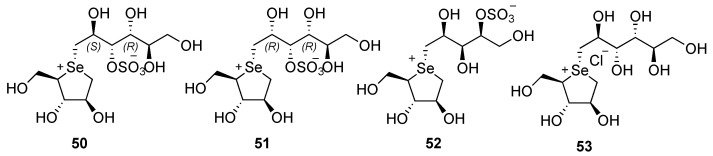
Structures of selenosugars **50**–**53**.

Considering the good activity of the C-7 extend chain derivative kotalanol (compound **45**, Figure 4) attention has been dedicated to the synthesis of different C-5′, C-6′, C-7′ polyhydroxylated chain derivatives, in which also the stereochemistry of the substituents and the sulfonate group have been modified. In Figure 6 some selected examples are reported.

As a general trend, it can be concluded that the elongated chain does not significantly increase the interaction with the active site of the enzyme. The stereochemistry of C-2′ and C-4′ is pivotal for the activity, indeed only the (2*S*,4*R*)-isomer **50** is capable of inhibiting the enzyme. The shifting of the sulfonate group from C-3′ to C-4′ resulted in a less potent compound, while the de-*O*-sulfonated derivative **53**, showed an increased inhibition activity against C-terminal sucrase isomaltase [49,54,55].

**Scheme 8:**
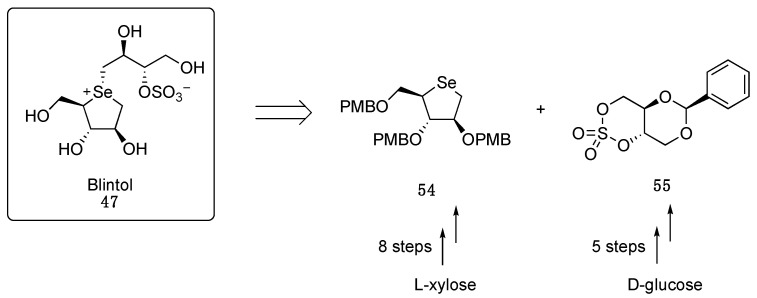
Retrosynthetic analysis of blintol (**47**).

Envisioning the need for a large amount of the potential drug candidate in a prospective clinical application, Pinto and co-workers developed an optimized, cost-effective, synthetic protocol able to yield a gram-scale synthesis of blintol (**47**). From a retrosynthetic perspective, compound **47** can be achieved by linking the two key syntones, namely 2,3,5-tri-*O-p*-methoxybenzyl-1,4-anhydro-4-seleno-*D*-arabinitol (**54**) and 2,4-*O*-benzylidene-*L*-erythritol-1,3-cyclic sulfate (**55**), the latter can be in turn prepared in five steps starting from D-glucose instead of the more expensive L-glucose (Scheme 8) [56].

**Scheme 9:**
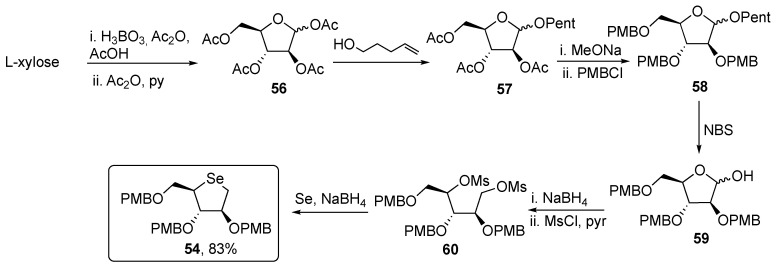
Synthesis of **54**.

Focusing on the synthesis of intermediate **54**, that is displayed in Scheme 9, there are several points of interest, such as the proper choice of the protecting groups, for example, *p*-methoxybenzyl instead of benzyl ether, which can be easily removed under mild acid conditions; moreover, the use of boric acid in the first step allows to push the equilibrium toward the formation of the furanoside structure, giving access to the protected derivative **56** in a one pot, two steps procedure. A careful investigation was also made to select *n*-pentenyl as the selective protecting group of the anomeric-hydroxyl group in compound **57**, which was then easily converted to compound **59**, using *N*-bromosuccinimide (NBS). Finally, the treatment with NaBH_4_, followed by the reaction with MsCl gave the activated intermediate **60**, that reacted with nucleophilic selenium generated in situ, affording the desired key intermediate **54** in a 20% overall yield [56].

## 4. Selenoglycosides

Selenoglycosides are a class of selenosugars that appeared in the literature in the early 1950s [57], in which the bridging oxygen of the glycosidic bond is replaced by selenium [58]. While their main biological property is the inhibition of glycosidase enzymes, which are unable to break the Se-glycosidic bond, they find synthetic applications in glycosylation procedures for oligosaccharides synthesis [59,60,61,62]. Selenoglycosides are used in crystallography thanks to the anomalous dispersion of selenium in response to X-ray irradiation; in this context, the methylselenoglycoside of *N*-acetylglucosamine was employed as a ligand mimetic for the structural determination of carbohydrate-binding protein F17-G adhesin [63]. More recently, the human galectin-9 *N*-terminal carbohydrate recognition domain was crystallized with the aid of selenosugars [64]. In the same context, dodecyl-β-selenomaltoside has been successfully utilized as a detergent for stabilizing membrane proteins in water [65].

**Scheme 10:**
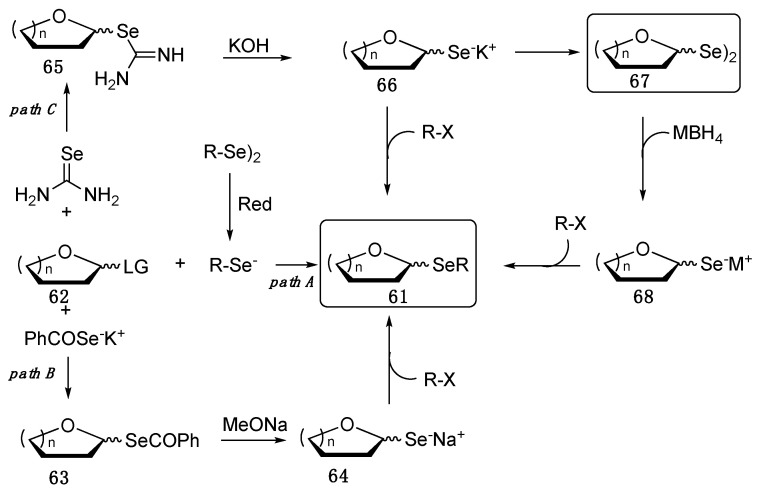
Synthetic approaches for the synthesis of selenoglycosides.

As reported in the general Scheme 10, selenoglycosides **61** can be prepared by installing the selenium at the anomeric carbon through a classic Koenigs–Knorr type procedure, where the chalcogen belongs to the aglycone that reacts with a protected sugar containing a leaving group (**62**, *path A*). Usually, the nucleophilic selenolate is used as such, as pioneeringly reported by Bonner and Robinson in the reaction of benzenselenol with peracetyl-α-d-glucopyranosyl bromide [57], and more recently by Di Bussolo and co-workers [66]. Alternatively, it is prepared in situ by the reduction of the corresponding diselenides, far more stable and easy-to-handle [67,68]. Examples of this kind of chemistry were reported by Misra, who has developed efficient, indium iodide and zinc-promoted Se-Se bond cleavage procedures [69,70]. These reactions proceed with inversion of the configuration at the anomeric carbon and, sometimes, it is not compatible with the protecting groups already present in the sugar.

Alternatively, selenoglycosides can be obtained following *path B*, where the leaving group-containing-protected sugar reacts with potassium selenobenzoate through, supposedly, a S_N_2 mechanism, affording compound **63** that, after the treatment with MeONa, gives the glycosilselenol **64**, which is a good precursor to prepare **61** and other derivatives [71]. The potassium analogue of **64**, compound **66**, can be prepared from the potassium hydroxide-promoted decomposition of the selenopseudoureido derivative **65**, that was in turn prepared through the reaction between selenoureas and **62** (*path C*). Interestingly, the potassium salt **66** was further evolved into the diselenides **67** [72], valuable compounds per se, but also precursors of nucleophilic (Scheme 10), electrophilic [73], or radical selenium-centered reagents [74]. Sugar diselenides were awfully prepared by Braga and Ludtke using Li_2_Se_2_ as selenylating agent [75,76].

**Scheme 11:**
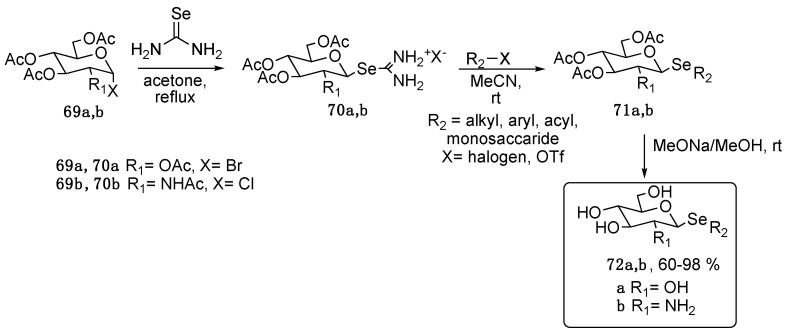
Synthesis of compound **72**.

A recent evolution of the *path C* methodology was reported by Kumar et al., who prepared selenonucleosides starting from commercially available, activated sugars (**69**, Scheme 11) and selenourea. The resulting isoselenuronium salts **70** were isolated and then functionalized with various alkyl, aryl, and acyl moieties or monosaccharides [77]. The authors prepared more than 30 compounds, all deprotected under basic conditions (compounds **72**).

**Scheme 12:**
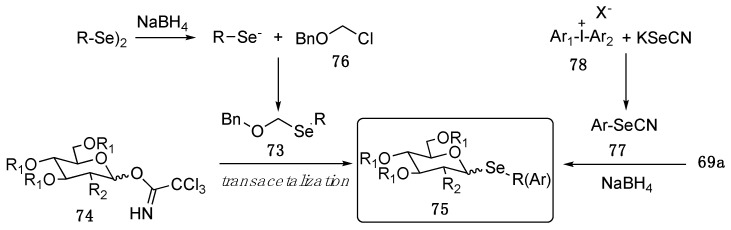
Approaches to selenoglycosides.

More recent approaches to prepare these selenocompounds are summarized in Scheme 12. Kiso and co-workers reported a transacetalization reaction between benzyloxymethyl alkyl selenide (**73**) and the glicosylimidate **74**, leading to the selenoglycosides **75** in high yields. The selenoacetals **73** were obtained by nucleophilic displacement of **76** with freshly prepared selenolates [78].

Very recently, Townsend and Guan reported the S_N_2 reaction between peracetylated α-glucosyl (**69a**) and α-galactosyl bromides and the selenolate formed in situ through the NaBH_4_ promoted reduction of arylselenocianates **77**. The reaction afforded stereoselectively the β−anomer as a result of the bimolecular nucleophilic displacement. Worth mentioning, the stated selenocyanates were smartly obtained by arylation of potassium selenocyanate using as electrophilic partner variously functionalized diaryliodonium salts **78** [79].

**Scheme 13:**
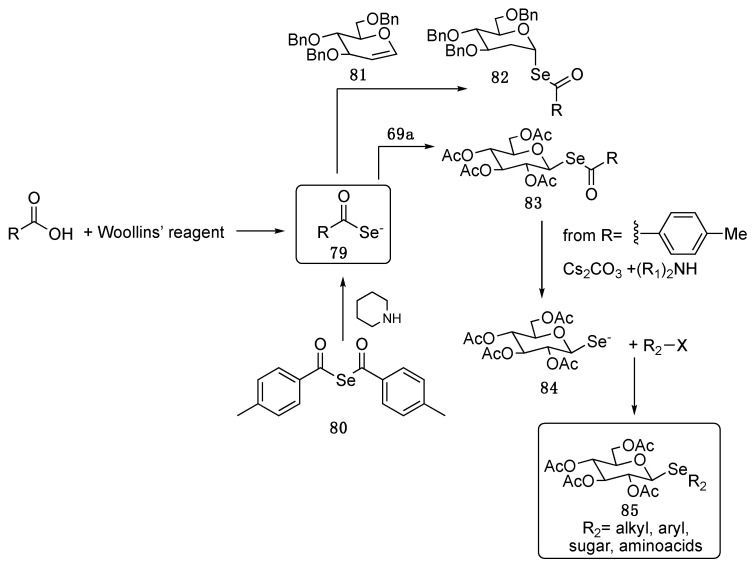
Selenocarboxylates in the synthesis of selenosugars.

Other exotic Se-centered nucleophiles used in the preparation of selenoglycosides are the selenocarboxylate anions **79**. They can be prepared by reacting carboxylic acids with Woollins’ reagent [80] or by the nucleophilic activation of *p*-methylselenobenzoic anhydride **80** [64]. Examples of their reactivities in the synthesis of selenoglycosides are reported in Scheme 13. Compound **79** adds to the benzyl protected glucal **81**, yielding cleanly and stereoselectively the α-adduct **82**. The nucleophilic displacement of the α-bromo-peracetyl-d-Glucose gave the protected β-selenosugar **83** [80]. The removal of the *p*-methylbenzoyl group is feasible and gives the selenolate anion **84**, that acts as synthetic handle to further modify the selenosugars with alkyl and aryl groups, or to prepare selenodisaccarides or, finally, aminoacyl decorated selenosugars [81]. Selenolactose derivatives, obtained through the above-mentioned methods, were used to solve the crystal structure of the human galectin-9 *N*-terminal carbohydrate recognition domain. Interestingly the X-ray structure of the selenosugar containing protein was almost the same of the lactose-containing one highlighting that the introduction of the heavier chalcogen did not affect the relative positions of either the protein or the ligands but made the analysis easier [64].

**Scheme 14:**
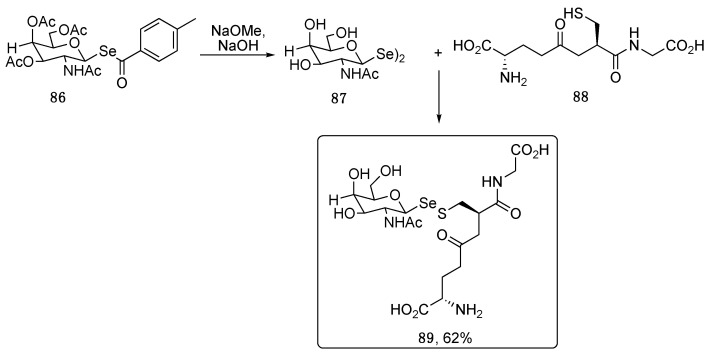
Synthesis of Hepatic Se metabolite A (**89**).

Using the piperidine-promoted activation of compound **80**, Davies and colleagues prepared the selenoester **86**, that was converted into the sugar diselenides **87** for the synthesis of glycoconjugated molecules bearing the unusual Se-S linkage (Scheme 14). Compound **89**, derived from the reaction between **87** and reduced glutathione **88**, is known as “Hepatic Se metabolite A”, which was prepared synthetically because it was just observed, but never isolated from living systems. More complex peptides were also decorated with selenosugars exploiting the same approach [82,83].

Compound **83** (R = *p*-MeC_6_H_4_, Scheme 13) was reported as inhibiting melanin synthesis in the context of a wider study aimed to identifying selenocarbohydrates endowed with the ability to modulate the melanin production in melan-a cells through the inhibition of tyrosinase, an enzyme which regulates the melanogenesis. The discovery of tyrosinase inhibitors is important for developing whitening products in both cosmetics and medicine [84].

**Scheme 15:**
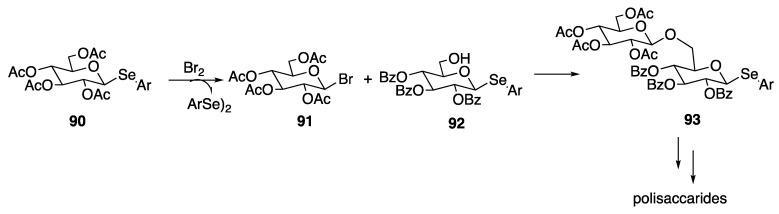
Synthesis of polysaccharides.

Selenoglycosides found application in oligosaccharide synthesis. As shown in Scheme 15, Yamago proposed a sort of armed and disarmed glycosylation strategy based on the ability of alkylarylselenides to react with molecular bromine affording the corresponding aryldiselenides and the sugar bearing a bromo group in the β-anomeric position. The installation of the bromo group served for the reaction with a second selenosugar, that acts as glycosyl acceptor thus forming the disaccharide **93**. Once formed, **93** undergoes activation with the bromo group and then reaction with further selenosugars in order to obtain polysaccharides [85]. This approach was later extended to the combinatorial synthesis of a library of oligosaccharides [86].

**Scheme 16:**
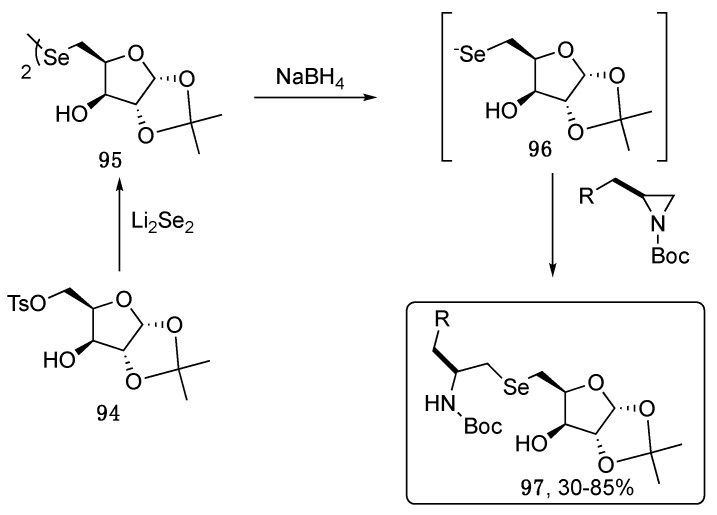
Synthesis of neoglycoconjugates.

Starting from diselenoglycosides, Braga and Ludtke reported the functionalization of selenolates formed in situ with protected aziridines, affording more complex selenocarbohydrates, named neoglycoconjugates [87]. As depicted in Scheme 16, the furanose derived diselenide **95**, prepared through the reaction of Li_2_Se_2_ and tosyl furanose **94**, was reduced to the corresponding selenol **96**, which was not isolated but rapidly reacted with variously functionalized, protected aziridines, giving the target compound **97**.

Pseudodisaccharides, where selenium links the two monosaccharides in a position different from the anomeric one, were also obtained [88,89] and the synthetic methods for their preparation were recently collected in a review article in which also their biological properties are discussed. Briefly, selenopseudo disaccharides were studied as lectin binders, that may have a number of potential therapeutic applications such as antibacterial (inhibitors of bacterial lectins), anti-inflammatory (selectins inhibitors), or anticancer (galectins inhibitors) agents [90].

## 5. Phosphoroselenoate Oligonucleotides

**Figure 7:**
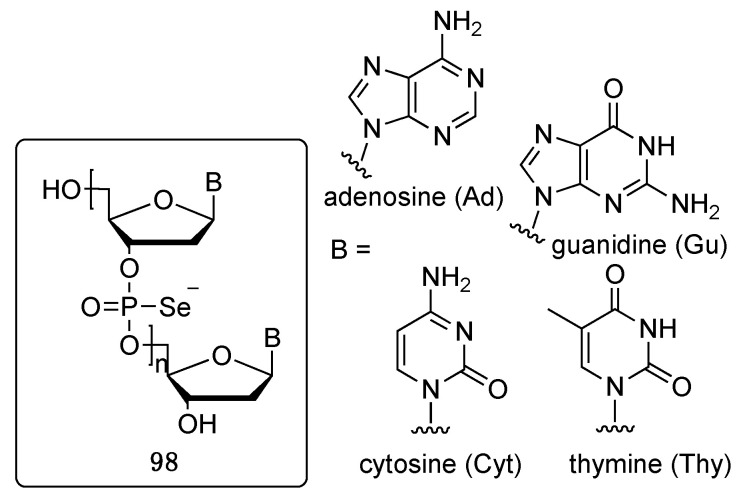
Structures of the phosphoroselenoates synthesized by Stec and studied by Mori.

Several papers and procedures in the literature report the synthesis and the biological properties of phosphoroselenoates, nevertheless, to the best of our knowledge, no review articles have covered this specific topic. The first report focused on the synthesis of phosphoroselenoate oligonucleotides (Figure 7) can be traced back to 1984, with the paper of Stec and colleagues [91]. Five years later, Mori et al. studied their biological properties as anti-HIV agents [92]. The synthetic methodology mainly relied in the late stage functionalization of phosphite intermediates with KSeCN, giving in general poor yields, even if in the context of an automated procedure [91].

**Scheme 17:**
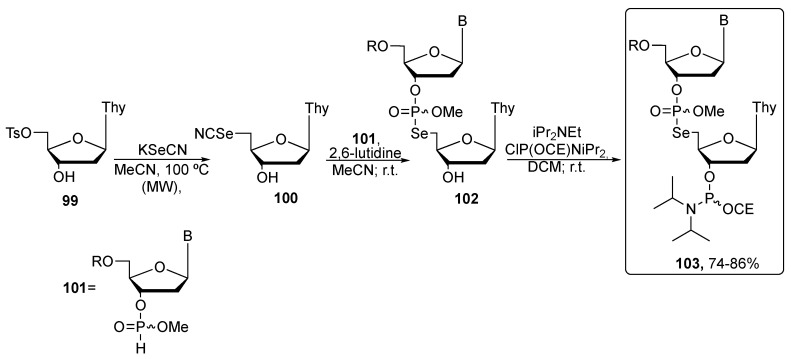
Synthetic pathway to obtain the phosphoroselenolates through Michaelis–Arbuzov reaction. R is 4,4′-dimethoxytrityl (DMTr) or 4-methoxytrityl (MMTr); CE: 2-cyanoethyl, and B is one of the DNA bases Cyt, Thy, Gu, Ad.

More recently, Conlon and co-workers proposed the synthesis of phosphoroselenolate oligomers using a multi-step protocol with better yields [93]. As shown in Scheme 17, the first step is the substitution of the tosyl group in the nucleoside **99** at the C-5 position by using an excess of potassium selenocyanate under microwave irradiation, in order to obtain the 5′-selenocyanate nucleoside **100** in 75% yield. The key step of the synthesis is the Michaelis–Arbuzov reaction of the methyl-protected nucleoside 3′-*H*-phosphonate **101** with compound **100**, that afforded the phosphoroselenolate-bridged dimer **102** with high efficiency. The presence of 2,6-lutidine served to improve the solubility of **100** in CH_3_CN, and all the dimers were obtained as diasteroisomers. Finally, the phosphitylation of the dimers allowed the preparation of the corresponding phosphoramidites **103**, which were then used as starting materials for the successive solid-state synthesis of oligomers, the actual target of these studies [93,94]. These selenium containing oligomers were used to observe conformational changes within nucleic acids during folding, recognition, and processing by applying the selenium single wavelength anomalous diffraction.

**Scheme 18:**
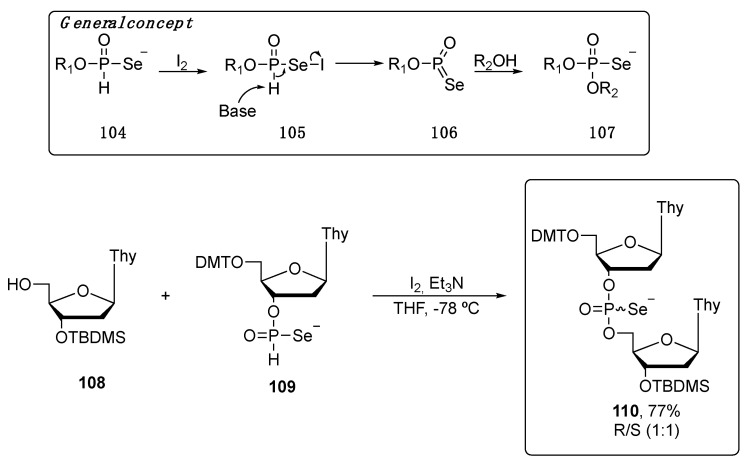
Oxidative pathway to obtain the phosphoroselenoates.

Alternatively, the phosphoroselenoate fragment can be obtained using *H*-phosphonoselenoate monoester **104** as starting material, as described by Bartoszewic et al. (Scheme 18). After an iodine-mediated oxidative pathway, **104** was converted to the intermediate iodoselenyl **105**, with a P(III). Under basic conditions, the phosphorus oxidation takes place with the concomitant elimination of iodide, forming the compound **106**. The target compound **107** was finally obtained by the treatment of **106** with water (R_2_ = H) or alcohols. The general concept was then applied to the synthesis of dinucleosides. In particular, compound **110** was prepared as a 1:1 diasteromeric mixture, as determined by ^31^P NMR, in 77% yield [95].

**Scheme 19:**
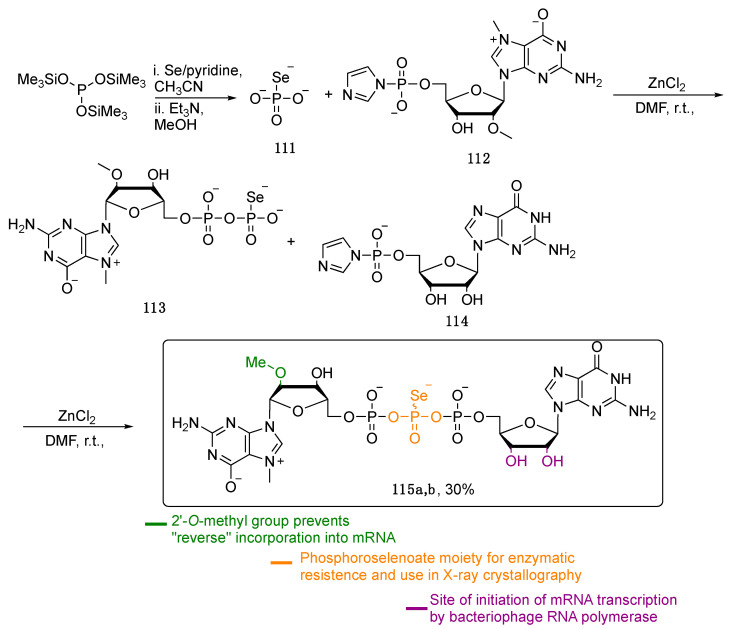
Pathway to obtain the asymmetric phosphoroselenoates **115**.

In 2009, Kowalska and co-workers reported the synthesis of asymmetric phosphoroselenolates, useful as synthetic RNA Cap analogue, and a valuable tool for studying mRNA translation and turnover. As reported in Scheme 19, the treatment of tris(trimethylsilyl)phosphite with elemental selenium in pyridine led to the unstable tris(trimethylsilyl)phosphoroselenoate **111** that, in the presence of the nucleoside **112** and an excess of ZnCl_2_ as Lewis acid, gives the more stable and easy-to-handle intermediate **113**. This latter was then converted into the target compound **115**, after its reaction with the imidazole-containing nucleotide **114**.

Compound **115** was obtained as a mixture of diasteroisomers, both submitted to biological evaluation for the binding affinities with the eukaryotic initiation factor 4E (elF4E), a protein responsible for rRNA Cap recognition during the initiation of translation. The presence of selenium generally stabilized the interaction with elF4E, and, as a result, compound **115** binds with higher affinity than the *O*-containing Cap analogues, and similarly to sulfur-containing ones. The mRNA sequences having incorporated compounds **115** at their 5′ends were efficiently translated, proving that the presence of phosphoroselenoates does not disturb the mRNA’s functionality [96]. These derivatives can be exploited to RNA-based gene therapy where mRNAs encoding viral- or tumor-associated antigens are transfected in the host dendritic cells triggering an autoimmunization process.

## 6. Se-Nucleosides

### 6.1. Selenium in the Sugar Backbone

Selenium-containing nucleosides are conceived as the next generation of nucleosides that may serve as starting point for the development of antiviral and anticancer drugs [97,98,99,100,101,102,103,104,105]. Indeed, the modification of the furanose ring has resulted in many marketed drugs [106]. To the best of our knowledge, the most recent example of 4′-selenonucleoside synthesis was reported in 2019 by Lee and co-workers, who described a procedure to obtain pyrimidine and purine 4′-selenonucleosides meant to act as anti-HCV agents [107].

**Scheme 20:**
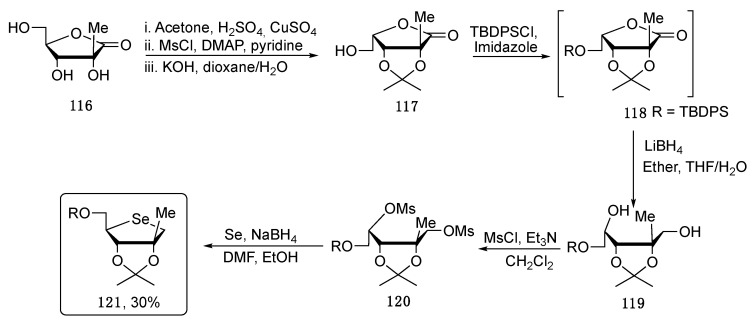
Synthetic pathway to obtain the selenosugar **121**.

As reported in Scheme 20, the authors have chosen the 2-C-methyl-D-ribono-γ-lactone **116** as starting material that, in a three steps telescopic reaction, was converted into the protected lactone **117** with an inverted configuration at the C-4. In the next step, **117** was protected at the primary alcohol with TBDPSCl, generating the intermediate **118**, which was subjected to a ring-opening reaction under reductive conditions to give **119**. In the sequence, the diol **119** was activated as mesylate and subjected to double nucleophilic substitution with freshly prepared Na_2_Se, leading to **121** in fairly good overall yield.

**Scheme 21:**
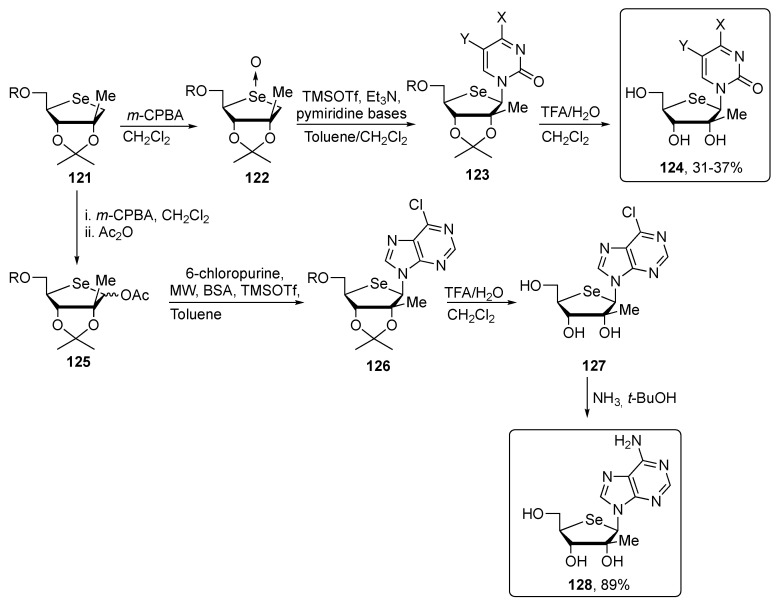
Synthetic pathway to obtain the selenosugars **124** and **128**.

With the protected selenosugar synthon **121** in hands, it was converted in both pyrimidines and purines nucleosides. The pyrimidine analogues **124** were prepared converting the selenosugars into a glycosyl-donor (**122**) and then decorated with the base through the Pummerer reaction [108], affording the compounds **123**, which were deprotected under acidic conditions. The target purine compound **128** was prepared converting the synthon **121** into the acetoxyl derivative **125**, through a Lewis acid catalyzed Vorbrüggen condensation. Compound **125** was then functionalized with 6-chloropurine, deprotected and aminated at the C-6, affording the target selenosugar **128** (Scheme 21).

In the same work, the authors converted purine and pyrimidine derivatives into phosphoramidate prodrugs, in order to obtain products more likely to have anti-HCV activity. Unfortunately, none of the synthesized compounds showed appreciable antiviral activity.

The big issue associated to the replacement of oxygen with selenium in the furanose ring is that the resulting nucleoside cannot be converted into the corresponding nucleotide because the cellular kinase is sterically hindered by the presence of the bulky selenium with its 4p orbitals, and the phosphorylation at the 5′ position is hampered [99,100]. As a result, such compounds lack biological activity. Among the exceptions, a selenonucleoside (LJ-2618) developed by Lee and colleagues, and biologically profiled in 2018, showed antitumoral potential in vivo and in vitro against paclitaxel-resistant prostate cancer cells. The plausible mechanisms by which LJ-2618 inhibits the growth of cancer cells may be a Skp2-dependent p27 stabilization [109].

**Scheme 22:**
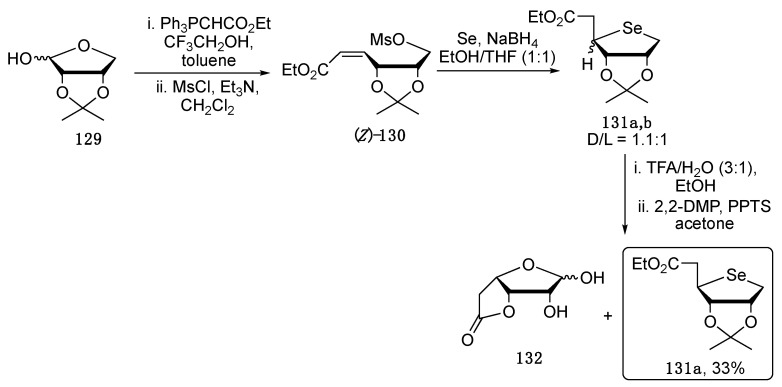
Synthetic pathway to obtain the key selenosugar intermediate **131a.**

In order to relieve the steric clash which prevents the cellular phosphorylation, Sahu and co-workers developed a synthetic route to obtain one carbon homologue of 4′-selenonucleosides, namely 5′-homo-4′-selenonucleosides with improved antiviral activity, thus better substrates of cellular kinases when compared to the regular 4′-selenonucleosides. The synthetic route, depicted in Scheme 22, started with the Wittig reaction on the 2,3-*O*-isopropylidene-l-erythrofuranose **129**, followed by the activation of the OH group as mesylate, affording **130** in a mixture with its *E*-isomer. In the presence of sodium selenide, **130** undergoes concomitant mesyl displacement and intramolecular Se-Michael addition, affording **131** in good yield but as a D/L mixture. Smartly, to isolate the target D compound **131a**, the D/L mixture was hydrolyzed with aqueous TFA and then treated with 2,2-dimethoxypropane in the presence of p-TsOH. In this way, the undesired L diasteroisomer was separated and discarded as the lactone **132**. In the same article, efforts were made to shift the stereoselectivity toward the D-isomer, by placing bulky groups such as TBS or TBDPS on the 2′ and 3′ positions of the starting sugar obtaining the target D compound in fourfold excess with respect to the undesired one [110].

**Scheme 23:**
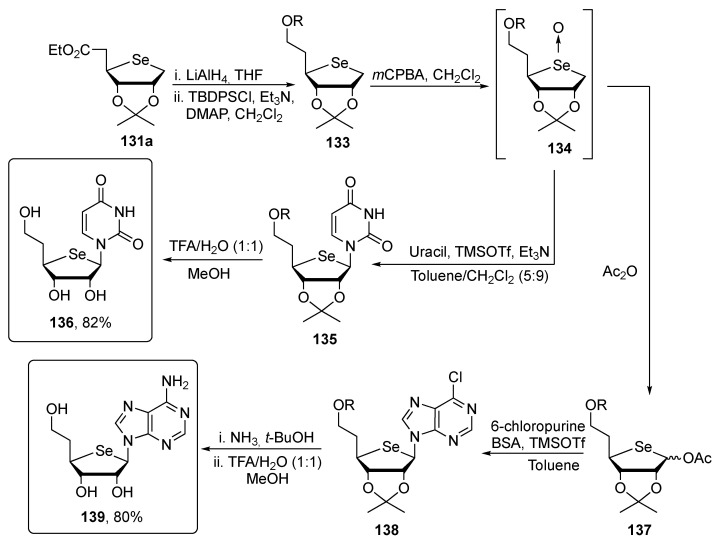
Synthetic pathway to obtain the 5′-homo-4′selenonucleosides **136** and **139**.

With the desired selenosugar **131a** in hands, the target carbon chain-extended nucleosides **136** and **139** were prepared by an initial lithium aluminum hydride reduction of the ester and protection with the tert-butyldiphenylsilyl group (TBDPS) yielding compound **133** that was converted into the key glycosyl-donor **134** by oxidation with *m*-CPBA. The intermediate **134** was condensed with the uracil base, and finally deprotected to afford **136** or, alternatively, it reacted with acetic anhydride to give the key synthon **137**, that was condensed with silylated chloropurine and finally deprotected to obtain the compound **139** (Scheme 23). The biological evaluation of compounds **136** and **139** clearly revealed that the homologation beneficially influenced the pharmacological properties, indeed they were far more potent than the corresponding 4′selenonucleosides when tested against human herpes simplex virus (HSV-1). Indeed 5′-homo-4′-selenonucleosides **136** and **139** displayed anti-HSV-1 activity in the low micromolar range (EC_50_ = 2.3 and 2.9 μM, respectively) whereas the corresponding 4′-selenonucleosides were completely inactive [110].

### 6.2. Selenium in the Nucleobase

Nucleosides bearing Se-containing bases were prepared and incorporated in oligonucleotides in order to study the pairing capabilities of the strains. One pioneering example is the one reported by Huang et al., who prepared 2-Se-uridine, its incorporation into oligonucleotides, and the investigation of their pairing capabilities [111].

**Scheme 24:**
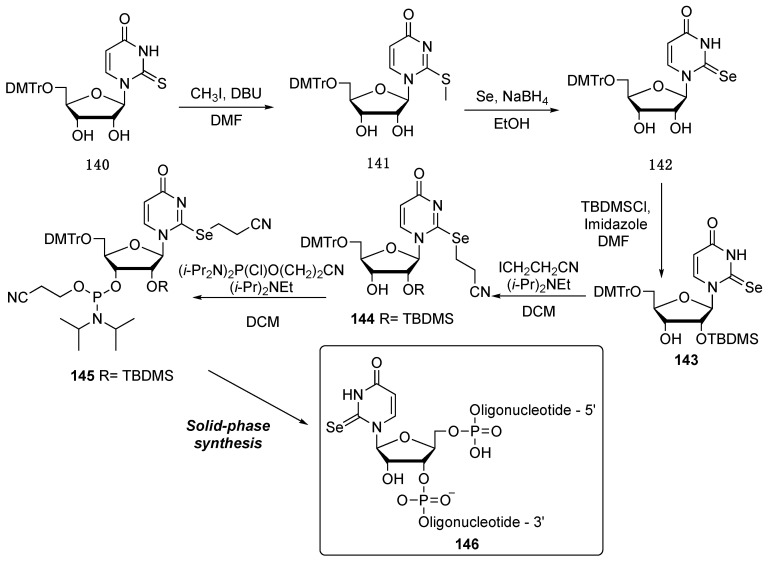
Synthetic pathway to obtain selenouridine-containing oligonucleotides.

For the synthesis of the desired RNA oligonucleotide incorporating the selenouridine nucleoside, the authors used as starting material the sulfur-containing nucleobase nucleoside **140**, that was methylated to promote the substitution reaction with the NaSeH, generated in situ, to obtain the seleno-uridine nucleoside **142**. Successively, the C-2 OH group was protected as TBDMS in the derivative **143**, unfortunately with low chemoselectivity. The selone functional group was then functionalized with a cyanoethyl moiety that permitted the successive incorporation into the nascent RNA oligonucleotide **146**, through solid phase synthesis (Scheme 24).

Additionally, Braga contributed to this field by preparing uridine derivatives bearing chalcogen in the C-5 position [112], while Knapp reported an electrophilic aromatic selenylation for the synthesis of 5-uridinyl derivatives then tested for their ability to inhibit malarial orotate phosphoribosyltransferase, a key enzyme in the Plasmodium falciparum life cycle [113].

**Scheme 25:**
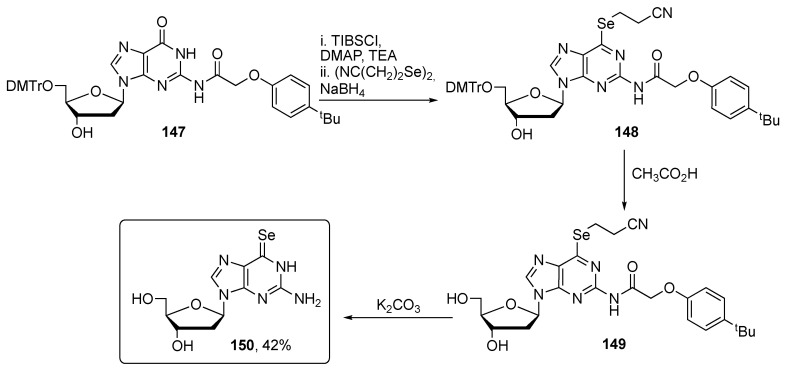
Synthetic pathway to obtain selenoguanine.

Examples of the selenium incorporation into purine nucleobases can be found in the works of Huang and co-workers. They prepared compound **150**, useful for investigating nucleoside transportation, nucleic acid–protein interaction, and nucleic acid folding, and studied the optical properties as well as its crystal structure [114,115]. The preparation entails first the introduction of the cianoethylselenyl moiety into the purine scaffold by aromatic nucleophilic displacement with cianoethylselenol, prepared in situ through the reduction of the corresponding diselenide. Compound **148** was then converted into the target selenonucleoside **150** via a two steps deprotection (Scheme 25).

**Scheme 26:**
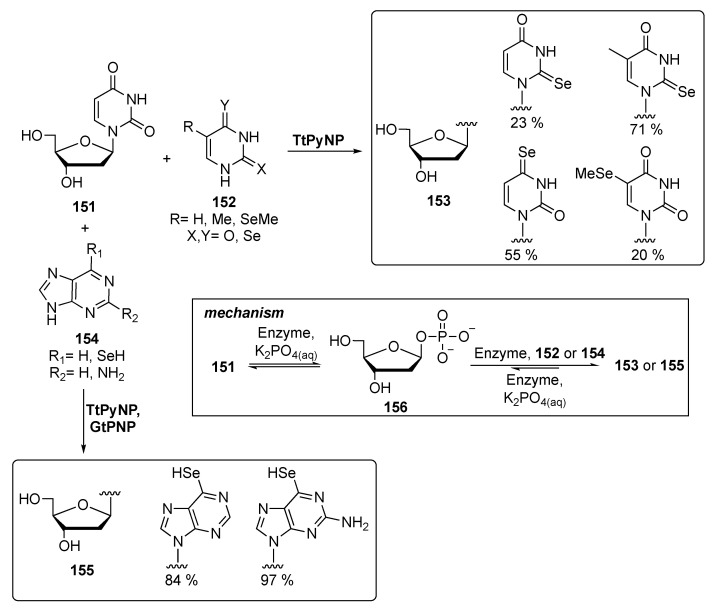
Biocatalytic approach for the synthesis of selenonucleosides.

Despite the advantages offered by the introduction of Se-containing nucleotides into nucleic acids, their synthesis is often complicated by the preparation of the selenium-containing nucleobase. As shown in Scheme 24, at least five steps are needed for the preparation of Se-uridine, with low overall yield. In an attempt to solve this issue, in 2019 Huang’s group proposed a new, biocatalytic method to obtain selenonucleosides [116]. This new strategy entails the combined action of two enzymes: pyrimidine nucleoside phosphorylase from *Thermus thermophilus* (TtPyNP), and purine nucleoside phosphorylase from *Geobacillus thermoglucosidasius* (GtPNP), which are involved in a transglycosilation reaction. Pyrimidine derivatives were prepared using TtPyNP as enzyme and urididine as furanose donor and a 1:5 molar ratio of selenium-containing nucleobases in phosphate buffer, while the purine derivatives were achieved through the tandem action of both TtPyNP and GtPNP (Scheme 26). Of course, the reactions have been carried out in aqueous solution at 60 °C without the need of organic solvents.

Noteworthy, the resulting nucleotides being negatively charged, all the reactions were followed by capillary electrophoresis analysis.

## 7. Conclusions

In this minireview, the selenosugars and the selenosugar-containing classes of compounds were analyzed in order to give fruitful insights to those interested in this research field or who are just approaching it. Different synthetic pathways were analyzed highlighting, where possible, the pros and cons and suggesting useful alternatives.

SeTal, compound **20**, is a clear example of an organoselenium compound endowed with antioxidant activity not only in vitro but also in vivo thanks to its water-solubility, which makes it suited for such kinds of studies. Selenonucleosides in which selenium is embedded in the sugar ring structure do display sufficient water-solubility but their biological activity is poor due to the lack of activation by cellular kinase. This issue was partially relieved by the synthesis of Se-homonucleosides. Finally, selenonucleosides were also detailed from a synthetic and applicative standpoint. In the author’s opinion, the spread of knowledge regarding this specific research field could help researchers to find solutions for the problem of the low aqueous-solubility, which hampers the actual clinical exploitability of many organoselenium compounds.
